# Waiting for other people: a psychoanalytic interpretation of the time for action

**DOI:** 10.12688/wellcomeopenres.15959.1

**Published:** 2020-06-10

**Authors:** Jordan Osserman, Aimée Lê

**Affiliations:** 1 Psychosocial Studies, Birkbeck University of London, London, UK; 2 English, University of Exeter, Penryn, UK

**Keywords:** psychoanalysis, Lacan, coronavirus, waiting, time, passivity, NHS

## Abstract

Typical responses to a confrontation with failures in authority, or what Lacanians term ‘the lack in the Other’, involve attempts to shore it up. A patient undergoing psychoanalysis eventually faces the impossibility of doing this successfully; the Other will always be lacking. This creates a space through which she can reimagine how she might intervene in her suffering. Similarly, when coronavirus forces us to confront the brute fact of the lack in the Other at the socio-political level, we have the opportunity to discover a space for acting rather than continuing symptomatic behaviour that increasingly fails to work.

## Introduction

‘ *A strike is precisely that kind of rapport that connects a group to work.’*

( Lacan, 2006, p. 266)

We were on the picket lines when the UK woke up to the reality that responding to COVID-19 was going to require mass shut-downs. We had been thinking and speaking, in University College Union ‘teach outs’, about how participation in industrial action opens up a particular and generative kind of temporal space. Withdrawing one’s labour dramatically disrupts the ‘on-go’ of daily life. One is thrown into a situation where time takes on a different quality: our relationship to the past is called into question (‘What has brought me to the point? Where have I been placed within the economic structure?’), and we gain a new sense of agency over the future through a rearticulation of the self. We thought this had something in common with the scenario of a patient undergoing psychoanalytic therapy, and we were attempting to tease out relevant parallels. 

This was the beginning of theorising an aspect of the psychic life of time rooted in a joyful form of collective struggle. It came to a dramatic halt with COVID-19, which suspended and indefinitely postponed strike action, while simultaneously throwing the causes for the dispute into sharp relief. What will happen to precarious staff employed on hourly and temporary contracts about to expire, accustomed to regularly moving across the country (or indeed the world) for insecure academic work, in the context of a pandemic and economic crash? How will university pensions, held in investment portfolios, endure a stock market freefall? Will we be told, yet again, that ‘now is not the time’ for rectifying the BAME and gender wage gaps, and that taking on unsustainable workloads in the shift to online teaching are simply part of being a team player during a ‘chaotic time’?

Neoliberal economics has shaped our healthcare provision (and indeed our health) for decades, ever since the introduction of ‘internal markets’ to the NHS, but the extent which health has been deprioritised in order to create an ‘efficient’ and profitable health service is now showing its true face. Prior to the outbreak, hospital occupancy had repeatedly hit all-time record highs, routinely exceeding 95% of capacity, leading 92% of doctors in a BMA survey to say that the NHS is ‘in a state of year-round crisis’ ( BMA, 2020) ^1^. The doctrine of profitability means no margin of ‘waste’ — which means no ability to cope with everyday volumes of patients, much less an actual crisis. It has become increasingly clear that our physical health relies not only on epidemiology but on the questions of politics, economics and analyses of social life more traditionally associated with the humanities and social sciences. The boundary between the physical and the social body has fallen. Here, we attempt to offer some suggestions with regard to these extraordinary times.

Concomitant with widespread fear of illness and economic ruin associated with COVID-19, we have observed the emergence of an unusual form of optimism. As governments around the world begin to implement stimulus and rescue packages designed to mitigate the economic effects of the disease — associated in the popular imaginary with wartime spending measures — some are beginning to hope that if we simply ‘wait’ (or ‘hang tight’) under quarantine, the government will ensure that things will be ‘okay’. Things will ‘return to normal’ eventually (as if returning to the state of affairs that gave rise to this crisis would be desirable), or even (in its more left-wing formulation), with the advent of socialist spending, a new and more equitable social order will arrive.

Keeping the racial implications of waiting in mind, we might remember colonial injunctions that the time was never right (so colonial subjects had to wait) for independence ( Chakrabarty, 2009), or in the US context, for emancipation and subsequently civil rights, about which Langston Hughes wrote a lyric to the ‘Hesitation Blues’: ‘How long/ have I got to wait?/ Can I get it now—/ Or must I hesitate?’ ( Hughes, 2001, p. 91).

So do we wait for these conclusions to sink in? This is a question of time, and also, clearly, a question of power. It is evocative of an early experiment in behavioural psychology, the Stanford ‘marshmallow experiment’, meant to explore the connection between delayed gratification and later successful life outcomes ( Mischel & Ebbesen, 1970). In the experiment, children were given the choice between an immediate reward (a marshmallow or pretzel), or two rewards if they were willing to wait for 15 minutes. The study, and subsequent others like it, linked children who waited with better test scores, better jobs, even better bodies ( Casey *et al.*, 2011; Mischel *et al.*, 1972; Shoda *et al.*, 1990). In mass media, the results of the study were promoted as a kind of neo-Calvinist doctrine of the persevering rich, as well as providing a handy economic allegory about the importance of obedience and trust when facing apparent deprivation. If you follow the rules (and don’t, for example, hoard toilet paper), the second marshmallow will be coming along any second now…

## The big Other falls apart

The researcher who dispenses the marshmallows is playing a role known psychoanalytically as the ‘big Other’ ^2^. As theorised by Lacan, the big Other stands for the place from which people imagine that authority ultimately emanates, a kind of ‘necessary illusion’ that grounds the otherwise potentially infinite uncertainty of subjective speech and behaviour. (‘The Other must first of all be considered a locus, the locus in which speech is constituted’ [ Lacan, 1997, p. 274].)

Individuals take on the mantle of the big Other insofar as they successfully appear to be a guarantor of futurity: my hands hold the keys to your fate. This is a structural relation between parent and child which, although eventually surmounted to varying degrees, becomes ‘transferred’ onto figures of authority actual and spectral.

However, as Derek Hook clarifies, ‘we should not fix the Other in any one personage, or view it in a static way as embodied in certain lofty or powerful figures. … We as subjects constantly call upon, reiterate and thus reinstate the Other … [it] is a (trans)subjective presupposition which exists only insofar as *we act as if it exists*’ ( Hook, 2017, p. 23).

Consider the way investors are speaking about ‘ the market’: ‘the market right now is really shellshocked’; ‘until the market sees some evidence that we’ve got the virus under control ... there isn’t going to be a lot of confidence to buy’. This anthropomorphic creature we call ‘the market’ is, of course, the sum total of individual investors’ financial behaviour. Yet, these investors do not decide whether to buy or sell stocks based directly on what they think other investors will do, but through the mechanism of a presupposed, transubjective third: *what I think other people think ‘the market’ is going to do* (see Tuckett, 2011).

In his late teaching, Lacan made a crucial emphasis on the notion of a *lack* in the big Other. At certain pivotal moments, we begin to realise that nobody is actually behind the curtain. The ‘glue’ that holds together a social order starts to melt.

The COVID-19 crisis is, of course, a prime example of such a moment. It is difficult to overstate just how incompetent and incoherent our political leaders have made themselves out to be. From Boris Johnson boasting that he was shaking hands with COVID-19 patients before contracting the virus ( The Guardian, 2020); to the government denying that it promoted ‘herd immunity’ ( Walker, 2020); to cabinet ministers openly contradicting WHO guidance in order to obscure the government’s failure to procure adequate testing, hospital equipment, and PPE ( ITV News, 2020) – it has become clear that there no longer exists a stable authority upon whose pronouncements we can rely (see especially recent exposes in the Guardian [ Conn *et al.*, 2020] and Sunday Times [ Calvert *et al.*, 2020]).

One of the ways Lacanian psychoanalysts approach the question of diagnosis is to consider how a patient responds when he is confronted with a lack in the big Other. Similarly, with the void in power that has emerged as a consequence of COVID-19, we are witnessing a variety of what we might call ‘symptomatic’ responses that index the coordinates of individuals’ psychic structures:

- Denial: the big Other is perfectly intact. *The novel coronavirus isn’t any worse than the ordinary flu, people are needlessly panicking due to social media and liberal commentators intent on discrediting our political leaders.*
- Conspiracy: we are being duped, a malevolent big Other is pulling the strings. *China designed COVID-19 as a biological weapon to destroy the West.*
- Deferral: give the big Other some time, and it will reconstitute itself. *Things are messy now, but if we just wait it out, they will return to normal. Once the government secures enough antibody tests, we can go back to work, the pubs will reopen, our holidays abroad will resume*.
- Panicked incapacitation: without the big Other, we are doomed. *The government is sending us all to our deaths and nothing can be done.*

In different ways, each of these responses indicate an attempt or wish to shore up the big Other, to retrieve some kind of guarantor of the body politic in the midst of its apparent breakdown ^3^.

Here we might also consider how a depoliticised portrayal of ‘Science’ itself constitutes a kind of ‘big Other’. Much of the government’s answer to criticism has been to claim that they are ‘responding’ to the ‘latest’ scientific findings and modelling -- effectively obfuscating the question of *which* scientists are being listened to and why, and ‘passing the buck’ for what are ultimately political decisions (see Scientific Advisory Group for Emergencies committee member Professor Graham Medley’s comments on this in Conn *et al.*, 2020). As Richard Horton, Editor of The Lancet, lamented in The Guardian (2020), ‘medical and scientific advisers to the UK government ignored [the] warnings’ of the Chinese scientists who published their findings regarding the ‘pandemic potential’ of COVID-19 in the Lancet on 24 January 2020. ‘For unknown reasons’ Horton writes, ‘[the UK government advisers] waited. And watched’ ( Horton, 2020). (See also John Ashton’s criticism of the pool of the government’s scientific advisers, ‘narrowly drawn … from a few institutions’, [ Grey & MacAskill, 2020])

As the clinician Thomas Svolos notes, ‘If psychoanalysis has something to offer here, it is to recognize ... the proper place of the lack in the Other, and the very personal nature of the fantasies we make to cover over it, so that people can soberly address the unknown’ ( Svolos, 2020). 

In other words, there is another approach: proceeding with the understanding that the lack in the Other was there from the beginning.

## Things were always this bad

In a sense, we all knew this was coming.

People were already perceiving that nobody was properly in charge. Regularly we received dire warnings about the NHS: waiting times at record highs, hospitals operating beyond capacity. Yet our transference towards the NHS as a safe parental figure (or ‘brick mother’) seemed to persist: people continued to believe that when they fell ill the NHS would provide adequate care (see Baraitser & Salisbury, 2020; Moore, 2020, *Waiting in Pandemic Times*).

Similarly, as fixed-term academics, we’ve long known that universities are simply not offering enough permanent posts for the majority of academics to do their work securely in the sector. Yet as a group we nevertheless persist as if we’ll all eventually find the right job. (UCU’s qualitative study on casualisation found an ‘inability to project into the future’ one of the significant mental health consequences of precarious academic work [ Megoran & Mason, 2020, p. 20].)

Psychoanalytically, the practice of simultaneously accepting *and* rejecting a traumatic truth -- continuing to behave as if it isn’t true – is called disavowal, summarised in the phrase: ‘I know very well, but nevertheless’ ( Mannoni, 1969).

In our daily life before COVID-19, we were already constantly surrounded by pronouncements of apocalypse, post-history, crisis and collapse — but these were always warnings, as it were, from ‘within’ the current coordinates, as society as a whole appeared to continue as normal (see Flexer, 2020, this collection). We were both present during the California wildfires of 2018, and despite the massive loss of life and environmental destruction, economic activity continued as usual, with the occasional addition of masks, respirators and so on. This seems to be a model for the way our government initially hoped we would respond to coronavirus.

## There is an opening

Before COVID-19, appeals for redistributive policies were easily diffused with the familiar language of technocratic neoliberalism: ‘the numbers don’t add up’ ‘this is not how it works’, etc. The message was: ‘your material suffering, while regrettable, does not have any bearing on the immutable laws of the economy’. With the sudden emergence of massive government spending — as we were writing this, the government cancelled £13.3 billion pounds of NHS debt — we’re witnessing this logic disappear before our very eyes.

This suspension of daily economic activity and the seemingly iron-clad principles that upheld it, alongside the threat of the virus, has interrupted the circuitry that forced us to act as if the big Other existed, even when all available evidence indicated otherwise.

We began from the transformative potential of suspended time in strike activity, which relies on the conscious decision of workers to withhold our labour. Now we have entered a different kind of suspended time. From the collectivity of the strike, we have gone into self-isolation, imposed by the current crisis. These are also not mutually exclusive; workers as well as renters have seized this time to strike. In both cases, however, different kinds of suspended time produce an opportunity for the subject to consider her own agency in relation to the lack in the big Other. 

It’s common for a patient to seek out analysis because a feeling of enjoyment, or what Lacanians call ‘jouissance’, is somehow no longer available. This instability provides an opportunity to reconsider the relation to the Other. In the current moment, we have arrived at a kind of analytic situation through simply suspending the function of enjoyment. The stock market is crashing but of course in neoliberal capitalism what is also crashing is our jouissance. Our typical release valves — going to the pubs, shopping — are gone. Amazon is deprioritising shipping anything but ‘essentials’, only ‘key workers’ and urgent tasks allowed ^4^.

We actually have to live in a time that is supposed to be a ‘waiting time’ — subjectively experience it as our reality in the here and now.

## Towards a theory of the act

Lacan in 1968, famously criticised student activists for posing what he took to be their hysterical demands to the powers that be: ‘You want a Master. You will get one’ (see Frosh, 2009). The protests of ‘68 were an explosion of activity, which we could counterpose to today’s means of reinstating a powerful Other through passivity.

The *act*, as theorised in Lacanian psychoanalysis, has to be distinguished from ‘acting out’, or everyday action. The true act has such stakes that it simultaneously abolishes and transforms (in Hegelian terms, sublates) the symbolic coordinates of a given social order. So, how and when do we act?

First, we have to find a way of acting within the context of there being no big Other. This means our actions cannot be verified or guaranteed to succeed from the outset. Nor, however, can we rely on an authority to predictably stop or punish us in the way transgression is often intended. Acts will always appear to us as risks — serious ones. This is even true when they are the self-evidently ‘right things to do’ in retrospect.

The corollary to this lack of divine verification is that *the time to act never arrives*. Even as people fall ill with coronavirus, and are no longer waiting to potentially contract it, the question of what to do is not resolved, it is even intensified.

We can say that an act never emerges from nothing, but only appears to in retrospect. We must be careful not to fetishize a moment of rupture for its own sake, or, as Baraitser (2017) reminds us, to fail to account for the preexisting context of *endurance within an impossible situation* upon which any significant rupture depends (the pre-existing ‘state of year-round crisis’ in the NHS, for example, which has led to this point). These would be further forms of acting out.

Lastly, an act must be collective but each of us cannot wait for another to start it. Those of us advocating for radical emancipatory change cannot simply make our individual appeals to ‘socialism’ as a self-evident intellectual solution to the problems we face, but must directly intervene to build it and create our own vehicles of mass struggle. Only through action can we instate *a new symbolic situation*. We can envision the collapse of neoliberal capitalism — a system that literally cannot function in the present situation — but without an alternative we will remain in the same symbolic coordinates. People are already beginning to figure out ways of coordinating activity during lockdown without risking their health, as technology creates an opportunity for greater international solidarity.

The emergence of ‘mutual aid’ groups across the country is an example of people coordinating responses to the crisis in the absence of adequate government provision. It is a first step but, at present, relies on the voluntary goodwill of people able to share what little they have with each other. The next step would be recognising the production and planning of resources in society — those zones where our intervention was once strictly forbidden — and seizing our right to directly provision to people’s material needs rather than obeying market logic. (It is a consequence of attempting to act that one may come to embody the big Other. This is a very interesting problem and should be dealt with in a subsequent essay.)

We need to push our governments to value human life over economic gain, but we must also recognise that our own activity is what will make this possible, not the benevolence of a Prime Minister. Revisiting the period of post-war reforms that delivered the NHS should make this clear. While claiming to support the principle of a health service in theory, Churchill’s opposition voted against the establishment of the NHS over a dozen times, including at Second and Third reading. The NHS was founded despite strong opposition from the Tories and the right-wing press, both of whom now praise it as a national achievement ^5^. None of the institutions we rely on now – especially during this crisis – came about because they were handed down from above. They were formed through processes of social antagonism. This poses the question: Why do people today view themselves as outside of the historical process?

Attempting to pose these questions to ourselves as well, we decided to act, to directly engage with universities to demand two years’ extension of employment for all casualised staff: a #CoronaContract ( https://coronacontract.org/).

We have reached a point where continuing within the existing framework of society is no longer possible. The question is, will we desperately search for another way to shore up the big Other, relying on symptomatic behaviour even as it fails to work – or can we find a way to act? 

## Data availability

All data underlying the results are available as part of the article and no additional source data are required.

## Author information

Jordan Osserman is a Postdoctoral Researcher on the Waiting Times project, based in the Department of Psychosocial Studies, Birkbeck (University of London), and a clinical trainee with The Site for Contemporary Psychoanalysis.

Aimée Lê is an Associate Lecturer in Creative Writing at the University of Exeter, and a member of Royal Holloway’s Poetics Research Centre.

## Footnotes

^1^ Indeed, the latest NHS planning document suggested 92% bed occupancy as an aspirational target: ‘Systems and organisations will be expected to reduce general and acute bed occupancy levels to a maximum of 92%. This means that the long period of reducing the number of beds across the NHS should not be expected to continue’ ( NHS, 2020).

^2^ A more sophisticated iteration of the ‘marshmallow experiment’ suggested that children who have experienced food poverty and other forms of deprivation might choose not to ‘wait’ due to their understanding that the big Other’s promise of eventual abundance would likely prove hollow ( Calarco, 2018; Watts *et al.*, 2018).

^3^ As feminist and critical race studies engagement with psychoanalysis has highlighted, the way one imagines and relates to the big Other and its inconsistencies is mediated through history, symbolic inheritance, and structural positioning along multiple axes of difference including race and gender (e.g. Chistopher & Lane, 1998; Fanon, 2008; Mitchell, 2015; Spillers, 1996). Likewise, the fallout from COVID-19 has differential impacts; while it is beyond the scope of this piece to explore, it is important to emphasise that the consequences of this disease will exacerbate existing inequalities and forms of oppression.

^4^ This of course throws into question what is or isn’t considered essential (such as medical care for trans people). It also shows how the ‘essential’ is now being reshaped as a space of jouissance by big business and the state: We can see a key revision of the role of ‘unskilled’ labour which previously operated in a largely mystified way. Michael Bloomberg previously said farming and factory work require less ‘gray matter’ than modern technology jobs (“You dig a hole, you put a seed in, you put dirt on top, add water, up comes the corn” [ Moore, 2020]); now, everyone is clapping for the NHS, calling for ‘land armies’ of agricultural workers, trying to inject some cultural enthusiasm back into these sectors in lieu of adequately funding them.

^5^ For more on post-war nostalgia as a form of austerity narrative (particularly the ubiquitous ‘Keep Calm and Carry On’ posters and their connection with the 2008 financial crisis), see Owen Hatherley’s, 2016 book The Ministry of Nostalgia. On the role of social antagonism within the history of the NHS, see Stewart, 2002; Webster, 1990.
