# Supplementary material for: Waiting for other people: a psychoanalytic interpretation of the time for action
Source: Wellcome Open Res. 2020 Jun 10;5:133. [Version 1] doi: 10.12688/wellcomeopenres.15959.1 (PMC7438963; doi:10.12688/wellcomeopenres.15959.1)
Supplement: Peer review history

# Peer review: Reviewer response for version 1

**DOI:** 10.21956/wellcomeopenres.17503.r40033

**Published:** 2020-08-19

**Authors:** Yannis Stavrakakis

**Affiliations:** 1 School of Political Science, Aristotle University of Thessaloniki, Thessaloniki, Greece

Obviously, the effects of COVID-19 extend far beyond the biological domain. They encompass many biopolitical, psychosocial and (psycho)political aspects in addition to health and welfare *stricto sensu*. This paper attempts to map and illuminate in an innovative way some of these effects. In particular, special emphasis is placed on the collapse of guarantees, the confrontation with failures in authority this crisis involves (as crises often do), a rubric that merits broader discussion. 

 Such failures are traced and framed on a variety of levels (economics, power, time, psychic life, etc.) and are then cogently theorized – through Lacanian theory – as encounters with the so-called ‘lack in the Other’, meaning the various instances in which one is bound to feel and, perhaps, have the opportunity to register the cracks in the fantasmatic consistency and the ultimately arbitrary (contingent) foundations of our socio-symbolic order. 

 How are such encounters usually dealt with? And how were they negotiated within the context of the COVID-19 crisis? In other words, are we doomed to reproduce a Sisyphean struggle to cover over this lack, which continuously reappears? Perhaps psychoanalysis can point to an alternative type of agency beyond this vicious circle, enabling thus a different ethos of political acting.

 All in all, the paper deals with a highly original, topical and timely theme. It performs an analysis, which is simultaneously accessible and rigorous, straightforward and conceptually sophisticated (drawing on a very pertinent Lacanian apparatus). The argument is indeed challenging, ambitious, witty and to the point. Thus, the paper does contribute significantly to the state-of-the-art in this field and is bound to influence the ongoing public debate in revealing ways. What is particularly suggestive is the axis of temporality, which is highlighted at various turns of the argumentation.

Is the work clearly and accurately presented and does it cite the current literature?

Yes

If applicable, is the statistical analysis and its interpretation appropriate?

Not applicable

Are all the source data underlying the results available to ensure full reproducibility?

No source data required

Is the study design appropriate and is the work technically sound?

Yes

Are the conclusions drawn adequately supported by the results?

Yes

Are sufficient details of methods and analysis provided to allow replication by others?

Yes

Reviewer Expertise:

psychoanalysis, Freud, Lacan, discourse theory, political theory, populism

I confirm that I have read this submission and believe that I have an appropriate level of expertise to confirm that it is of an acceptable scientific standard.

# Peer review: Reviewer response for version 1

**DOI:** 10.21956/wellcomeopenres.17503.r39809

**Published:** 2020-08-05

**Authors:** Todd McGowan

**Affiliations:** 1 University of Vermont, Burlington, VT, USA

"Waiting for Other People" outlines the effect of the coronavirus pandemic on the contemporary political situation. It points out that one of the main effects of the outbreak is that it exposes the lack in the Other or the failure of the big Other. Social authority is unable to deal with the disease, and as a result, subjects' investment in the figure of the big Other comes into question. The most widespread response, the authors claim, is the attempt to shore up the big Other, to obscure its lack. But at the same time, the virus presents us with another opportunity - the possibility of the genuine political act that occurs through the Other's failure. 

 This essay represents an outstanding intervention in the psychoanalysis of the effects of the pandemic. I have read several psychoanalytic accounts of our political situation today, and this is the best. I don't view any changes as necessary.

Is the work clearly and accurately presented and does it cite the current literature?

Yes

If applicable, is the statistical analysis and its interpretation appropriate?

Not applicable

Are all the source data underlying the results available to ensure full reproducibility?

Yes

Is the study design appropriate and is the work technically sound?

Yes

Are the conclusions drawn adequately supported by the results?

Yes

Are sufficient details of methods and analysis provided to allow replication by others?

Yes

Reviewer Expertise:

dialectics, psychoanalysis

I confirm that I have read this submission and believe that I have an appropriate level of expertise to confirm that it is of an acceptable scientific standard.
